# Study of phylogenetic relationship of Turkish species of *Klasea* (Asteraceae) based on ISSR amplification

**DOI:** 10.3897/phytokeys.56.5608

**Published:** 2015-09-24

**Authors:** Bekir Dogan, Ahmet Duran, Meryem Şeker, Özlem Çetin, Esra Martin

**Affiliations:** 1 Necmettin Erbakan University, A. K. Education Faculty, Department of Science Education, TR-42090, Meram-Konya, Turkey; 2 Selcuk University, Faculty of Science, Department of Biology, TR-42075, Selçuklu- Konya, Turkey; 3 Necmettin Erbakan University, Faculty of Science, Department of Biotechnology, TR-42090, Meram-Konya, Turkey

**Keywords:** Asteraceae, ISSR, *Klasea*, *Serratula*, Molecular systematics, Phylogeny

## Abstract

*Klasea* is a taxonomically complex genus in which there are many problems, mostly with *Klasea kotschyi* and *Klasea hakkiarica*. It is challenging to differentiate the genera based on morphological characters alone. Revision studies performed on the basis of molecular data obtained from studies conducted in recent years have made the phylogenetic relationships and systematic positions of the taxa more apparent and reliable. In this study, *Klasea*, *Serratula*, *Jurinea* and *Centaurea* species native to Turkey, were collected from different localities of Anatolia and DNA was isolated from the collected samples. The data were analyzed ordination analyses including UPGMA and PCA using NTSYSpc 2.1. The infrageneric and intergeneric phylogenetic relationships between *Klasea* and other related genera were also characterized. The *Klasea* species were grouped into three clusters. It was determined that taxa *Klasea kotschyi* and *Klasea hakkiarica* are separate but closely related. Moreover, it was observed that the *Klasea lasiocephala* a separate group within the genera. Clearly the genera *Klasea*, *Serratula*, *Jurinea* and *Centaurea* are phylogenetically differentiated on the dendogram.

## Introduction

The tribe Cardueae (Asteraceae) is generally accepted to be classified into five subtribes named Echinopinae, Carlininae, Carduinae, Centaureinae and Cardopatiinae (Susanna et al. 2006). Cardueae include perennial, biennial, or monocarpic herbs and shrubs and, less often, annual herbs or small trees (Barres et al. 2013). However, delineation of these taxonomic entities is highly problematic. Beyond the limits of the tribes, the boundaries between these units are also very difficult to establish. Also, some large genera of the tribes have generic delimitation problems: *Carduus* L. (90 species), *Cirsium* Mill. (250 species), *Centaurea* L. (400 species), *Cousinia* Cass. (800 species), *Serratula* L. (70 species), and *Saussurea* DC. (more than 300 species) (Garcia-Jacas et al. 2002). Extensive work conducted recently by Garcia-Jacas et al. (2000, 2001) and Font et al. (2002) have clarified the delineation of *Centaurea*. Limited studies also exist on *Cirsium* and *Carduus* (Haffner and Hellwig 1999), but most of the taxonomic problems persist. The genus *Klasea* Cass. constitutes a taxonomically complex group of plants with generic boundaries are unclear, especially at the generic level surrounding genus *Serratula* (Martins and Hellwig 2005). Klasea Cass., traditionally treated as a section within *Serratula* L., is widely accepted at the generic level (Martins 2006). *Klasea* is naturally distributed in Central Asia, Iran, Turkey, China, Himalayas, south east Europe and south Russia. *Klasea* is located within the monophyletic tribe Cardueae, in the subtribe Centaureinae (Susanna et al. 2006).

16 species were reported for the genus *Serratula* in Turkey (Davis and Kupicha 1975; Davis et al. 1988). Then all Turkish *Serratula* species were transferred to *Klasea* except *Serratula tinctoria* (Greuter 2003; Martins 2006). Thus, *Klasea* is represented by 15 species and *Serratula* is represented by one species within the Mediterranean and Irano-Turanian phytogeographic regions of Turkey (Dogan et al. 2012). Five of these species are endemic to Turkey, resulting in an endemism ratio of 33.3% (Dogan et al. 2012).

Currently, morphological revisions of various plant taxa are often supported by molecular data (APG 2003). As compared with morphological data, DNA sequences are not influenced by the environmental conditions in which the plants have grown; hence they serve as a powerful tool in resolving taxonomical and systematical problems. When compared with the phenotypic characters, by using different molecular marker systems, more reliable results were also obtained by a number of researchers that used different plant groups (Yang et al. 1996; Joel et al. 1998; Soranzo et al. 1999; Bremer et al. 2001; Mengitsu et al. 2002; Ash et al. 2003; Jump et al. 2003; Pharmawati et al. 2004; Dogan et al. 2007; Ali et al. 2013).

The RAPD (Randomly Amplified Polymorphic DNA) fingerprinting method is widely used and has a wide range of applications (Williams et al. 1990). However, because RAPD is a highly sensitive method, it should be used with great care. The ISSR (Inter Simple Sequence Repeat) has much higher levels of reproducibility than RAPD, for which reason it is preferable (Zietkiewicz et al. 1994, Prevost and Wilkinson 1999; Dogan et al. 2007; Hakki et al. 2010). The ISSR method is very widely used for the analysis of genetic diversity (Prevost and Wilkinson 1999).

Simple sequence repeats (SSRs), also known as microsatellites, are tandemly repeated di-, tri, tetra- or penta-nuclotide sequences (mainly within the range of 10–80 repeats of the core unit) that are abundant within eukaryotic genomes. A high level of genomic variation is generated by the more or less evenly distributed microsatellite sequences present within the plant and animal genomes. The high levels of genomic variation are widely used for genetic variation analysis of both wild plants (Wolfe et al. 1998; Dogan et al. 2010; Laosatit et al. 2013; Khalik et al. 2014) and crop plants (Vosman and Arens 1997; Hakki et al. 2001; Mohammadzadeh et al. 2011). Microsatellites can be used in inter- as well as intra-species analyses (Soranzo et al. 1999). However, the technique requires prior sequence information for the locus-specific primers, a feature that renders it difficult to be applied to plants for which no adequate genomic sequencing studies exist. Without considering their difficulty or cost (Hakki and Akkaya 2000), numerous microsatellite loci have been identified for economically important crops such as wheat, rice or maize. In *Klasea*, however, they have not been utilized.

In this study, *Klasea* species, which are difficult to delineate using morphological traits, were collected from their natural habitats in Turkey. DNA was isolated and fingerprinting was performed using a highly reliable and reproducible technique that mimics the application ease of RAPDs. The method employed to assess the genetic diversity and to resolve the genetic relationships among the species is a technique derived from SSR characterization based on PCR amplification of ISSR regions primed by a single oligonucleotide corresponding to the targeted repeat motif. The SSR-containing primers are usually 16-25 base pair long oligonucleotides anchored at the 3’- or 5’-end by two to four arbitrary, and often degenerate, nucleotides (Fang et al. 1997). The primer can be based on any of the motifs found at SSR loci. In these conditions, only sequence regions flanked by the two adjacent identical and inversely oriented microsatellites are amplified. Overall, the technique does not require prior sequence information (an advantage against microsatellites) and its reliability is higher than RAPD’s.

The aim of this study was to determine the genetic relationships among selected Anatolian-originated *Klasea*, *Serratula*, *Jurinea* and *Centaurea* species collected from diverse regions of Turkey and to use a DNA-based molecular marker system to resolve the unclear and controversial status of these species based on conventional morphological characters.

## Material and methods

### Specimen collection

Silica gel dried plant leaf samples belonging to 15 *Klasea* taxa and *Serratula tinctoria*, and 2 out-group taxa (*Jurinea* and *Centaurea*) were collected from the natural flora of Turkey. The species and provinces of their localities are as follows: *Klasea quinquefolia* (Artvin), *Klasea oligocephala* (Kahramanmaraş), *Klasea kotschyi* (Bitlis), *Klasea serratuloides* (Van), *Klasea erucifolia* (Erzurum), *Klasea lasiocephala* (Antalya), *Klasea cerinthifolia* (Kahramanmaraş), *Klasea grandifolia* (Antalya), Klasea radiata subsp. radiata (Kars), *Klasea hakkiarica* (Hakkari), *Klasea haussknechtii* (Muş), *Klasea coriaceae* (Kars), Klasea radiata subsp. biebersteiniana (Kars), *Klasea kurdica* (Osmaniye), *Klasea bornmuelleri* (Malatya), *Serratula tinctoria* (Bolu), *Centaurea ptosimopappoides* (Adana), *Centaurea straminicephala* (Erzurum), and *Jurinea cataonica*. For details see Table 1.

**Table 1.** List of sampled taxa. Including location data, collectors, and herbarium in which the voucher specimens are accessioned.

Species	Voucher
*Klasea serratuloides*	Turkey, Van: Van to Gurpinar, 2125 m, 38°24.434’N, 043°23.079’E, 19.07.2009, B.Doğan 2117 & A.Duran (KNYA).
*Klasea lasiocephala*	Turkey, Antalya, Gazipasa, Çayıryaka mountain pasture, 1730 m, 36°.30.027'N, 032°32.181'E, 30.06.2009, B.Doğan 2105 & A.Duran (KNYA), Endemic.
*Klasea bornmuelleri*	Turkey, Malatya, Darende, near Akçatoprak, 1010 m, 38°30.064'N, 037°33.907'E, 17.07.2009, B.Doğan 2110 & A.Duran (KNYA), Endemic.
*Klasea kurdica*	Turkey, Osmaniye, Yarpuz, 1465 m, 37°00.774'N, 036°26.683'E, 15.07.2009, B.Doğan 2106 & A.Duran (KNYA).
*Klasea coriaceae*	Turkey, Kars, Tuzluca to Kağızman, 1055 m, 40°06.399'N, 043°29.567'E, 20.07.2009, B.Doğan 2122 & A.Duran (KNYA)
*Klasea cerinthifolia*	Turkey, Kahramanmaraş, Ahir mountain, 990 m, 37°36.470'N, 036°52.917'E, 16. 07.2009, B.Doğan 2107 & A.Duran (KNYA)
*Klasea grandifolia*	Turkey, Antalya, Akseki, Süleymanlı village, 1425 m, 37°17.980'N, 031°46.520'E, 31.07.2009, B.Doğan 2130 & A.Duran (KNYA).
*Klasea haussknechtii*	Turkey, Muş, Malazgirt, Karıncalı village, 1840 m, 39°21.219'N, 042°20.010'E, 18.07.2009, B.Doğan 2113 (KNYA).
Klasea radiata subsp. biebersteiniana	Turkey, Kars, Kağızman, Akçay to Cumaçay, 1830 m, 20.07.2009, B.Doğan 2124 & A.Duran (KNYA).
Klasea radiata subsp. radiata	Turkey, Kars, Arpaçay, Kardeşköy to Dağköy, 2190 m, 06.08.2010, 40°55.087'N, 043°11.209'E, B.Doğan 2283 & A.Duran
*Klasea hakkiarica*	Turkey, Hakkari, Cilo mountain, Kırıkdağ, near dez stream, 2210 m, 37°32.974'N, 043°57.615'E, 07.08.2009, B.Doğan 2132 & A.Duran (KNYA), Endemic.
*Klasea kotschyi*	Turkey, Bitlis, Tatvan, Sapur village, 1965 m, 38°26.154'N, 042°24.413'E, 06.08.2009, B.Doğan 2131 & A.Duran (KNYA).
*Klasea quinquefolia*	Turkey, Artvin, Ardanuç, Boyalı village, 1210 m, 41°06.967'N, 042°07.283'E, 11.08.2009, B.Doğan 2139 & A.Duran (KNYA).
*Klasea erucifolia*	Turkey, Erzurum, Köprüköy, Eğirmez village, 1635 m, 39°57.056'N, 041°51.530'E, 09.08.2009, B.Doğan 2137 & A.Duran (KNYA).
*Klasea oligocephala*	Turkey, Kahramanmaraş, Ahir mountain, 995 m, 37°36.475'N, 036°52.947'E, 16. 07.2009, B.Doğan 2108 & A.Duran (KNYA).
*Serratula tinctoria*	Turkey, Bolu, Gerede to Bolu, 28. km, 1105 m, 09.08.2010, 40°45.340'N, 031°54.888'E, B.Doğan 2290 & A.Duran (KNYA).
*Jurinea cataonica*	Turkey, Erzincan, Old Çayırlı road, 10. km, 1750 m, 39°47.954'N, 039°30.343'E, 07.08.2005, B.Doğan 1029 (KNYA). Endemic.
*Centaurea ptosimopappoides*	Turkey, Adana,Aladağ to Kızıldag, 890 m, 19. 06. 2010, A.Duran 9042 & M.Öztürk (KNYA).
*Centaurea straminicephala*	Turkey, Erzurum,Uzundere to Artvin, 1100 m, 26.07.2002, A.Duran 6048 & M.Sağıroğlu (KNYA).

### DNA extraction

Nuclear DNA of silica gel dried leaf samples were extracted according to the instructions of the Nucleon phytopure plant DNA extraction kit (RPN 8510, Amersham Life Science, England). For each sample, DNA was extracted from 100 mg of leaf. After concentrations were determined using an Eppendorf BioPhotometer, DNA samples were diluted to the working concentration of 25 ng/µL. To better quantify the DNA and to assess the quality of the DNA, samples were run on an agarose gel (0.9%), stained with ethidium bromide, against a DNA standard with known concentrations. Stock DNA was kept at -86 °C.

### ISSR Amplifications

Of the 20 primers investigated during our initial screening, the primers that gave the most informative patterns (in terms of repeatability, scorability, and the ability to distinguish between varieties) were selected for fingerprinting. The characteristics of the primers used are given in Table 2.

**Table 2.** List of the ISSR primers used in this study and their specifications.

Primer	Primer sequence	T_m_ (°C)	Size (bp)	GC (%)	Number of polymorphic bands
ISSR F1	GAG CAA CAA CAA CAA CAA	49.1	18	38.9	13
ISSR F2	CTC GTG TGT GTG TGT GTG T	56.7	19	52.6	11
ISSR F3	AGA GAG AGA GAG AGA GCG	56	18	55.6	14
ISSR F4	AGA GAG AGA GAG AGA GTG	53.7	18	50	12
ISSR F5	AGA GAG AGA GAG AGA G	49.2	16	50	10
ISSR F6	CCA CCA CCA CCA CCA	53.3	15	66.7	13
ISSR F7	ACA CAC ACA CAC ACA C	49.2	16	50	12

Each reaction contained 2.5 mM MgCl_2_, 10 mM Tris-HCl (pH 8.8), 50 mM KCl; 0.8% Nonidet P40, 200 mM of each of dNTP, 0.5 mM primer, 25 ng DNA template and 0.4 units of Taq DNA Polymerase (Bioron, Germany) in a final reaction volume of 25 µl. After a pre-denaturation step of 3 minutes at 94 °C, amplification reactions were cycled 40 times at 94 °C for 1 minute, at annealing temperature (Table 1) for 50 seconds and 72 °C for one minute followed by a final 10 minutes 72 °C extension in an Eppendorf Mastercycler gradient thermocycler. Upon completion of the reaction, aliquots of PCR products (15 µL) were mixed with 3 µL of loading buffer (50% glycerol, 0.25% bromophenol blue and 0.25% xylene cyanol), loaded onto a 2.0% agarose/1x Tris-Borate EDTA gel and electrophoresed at 4 V/cm.

Amplifications were repeated at least twice at different time periods for each primer using the same reagents and procedures.

### Data collection and cluster analysis

Amplified fragments were visualized under a UV transiluminator and photographed using a gel documentation system (Vilbert Lourmat, Infinity model). All of the amplified fragments were treated as dominant genetic markers. Each DNA band generated was visually scored as an independent character or locus (1 for presence and 0 for absence). Qualitative differences in band intensities were not considered. Every gel was scored in triplicate (independent scorings) and only the fragments consistently scored were considered for analysis. A rectangular binary data matrix was prepared and all the data analysis was performed using the Numerical Taxonomy System, NTSYS-pc version 2.1 (Applied Biostatistic, Exeter Software, Setauket, New York, USA).

In cluster analysis of the samples, the unweighted pair-group method with the arithmetic mean (UPGMA) procedure was followed (Rohlf 1992). The genetic distances were calculated with the SM coefficient. In order to determine the ability of ISSR data to display the inter-relationships among the samples, principle co-ordinate analysis (PCA) of pair-wise genetic distances (Nei 1972) was also conducted using the NTSYS-pc package.

## Results and discussion

Silica gel dried plants collected from 19 different natural habitats were taken to the laboratory. The total number of species collected and used in the phylogenetic analysis was 19. DNA extractions were first attempted using a standard 2X CTAB method. Due to the poor DNA quality produced by the CTAB procedure, a commercial kit (Nucleon phytopure) was used in all isolations and repeated extractions were conducted whenever necessary.

**Figure 1.**
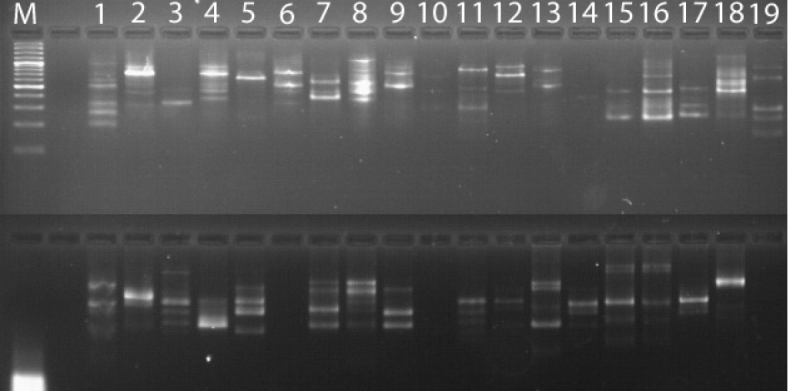
Representative agarose gels where PCR products were amplified with the primers ISSR F5 (highest number of polymorphic bands, top) and ISSR F5 (lowest level of polymorphic bands, down). **1** *Serratula tinctoria* **2** *Klasea quinquefolia* **3** *Klasea oligocephala* **4** *Klasea kotschyi* **5** *Klasea serratuloides* **6** *Klasea erucifolia* **7** *Klasea lasiocephala* **8** *Klasea cerinthifolia* **9** *Klasea grandifolia* **10** Klasea radiata subsp. radiata **11** *Klasea hakkiarica* **12** *Klasea haussknechtii* **13** *Klasea coriaceae* **14** Klasea radiata subsp. radiata **15** *Klasea kurdica* **16** *Klasea bornmuelleri* **17** *Centaurea ptosimopappoides* **18** *Centaurea straminicephala* **19** *Jurinea cataonica*, M: marker.

From an initial screening of 20 ISSR primers, seven primers revealed high levels of polymorphisms. These primers generated 85 highly polymorphic fragments that were consistently amplified in repeated experiments conducted on separate dates. The GC percentages of the selected primers were within the range of 38.8–66.7%. The characteristics as well as the sequences of the primers revealing a polymorphism are shown in Table 2. The primer ISSR F3 amplified the highest number of polymorphic fragments (14 bands) and primer ISSR F5 yielded the lowest number of fragments (10 bands). In total, the average number of polymorphic fragments per primer used was roughly 12. A representative figure containing ISSR F3 and ISSR F5 banding patterns is given in Figure 1.

**Figure 2.**
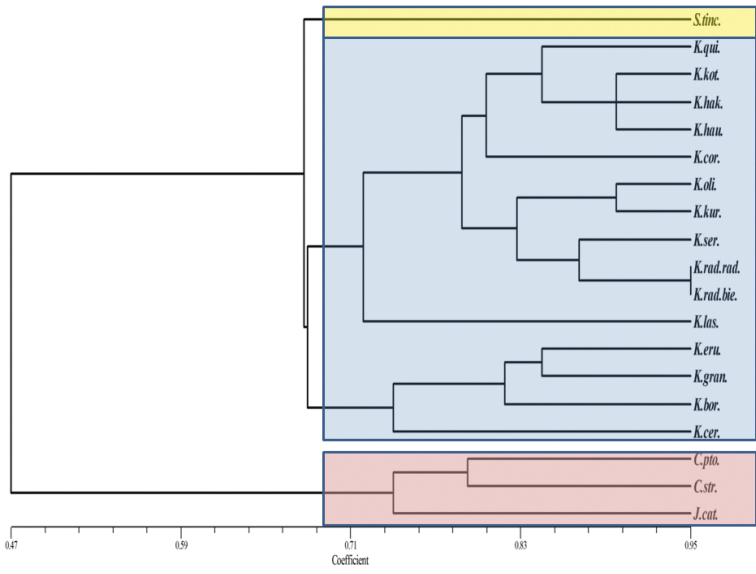
Dendrogram showing genetic relationship of *Klasea*, *Serratula*, *Centaurea* and *Jurinea* species as shown using inter simple sequence repeats. (*Serratula tinctoria*, *Klasea quinquefolia*, *Klasea oligocephala*, *Klasea kotschyi*, *Klasea serratuloides*, *Klasea erucifolia*, *Klasea lasiocephala*, *Klasea cerinthifolia*, *Klasea grandifolia*, Klasea radiata subsp. radiata, *Klasea hakkiarica*, *Klasea haussknechtii*, *Klasea coriaceae*, Klasea radiata subsp. radiata, *Klasea kurdica*, *Klasea bornmuelleri*, *Centaurea ptosimopappoides*, *Centaurea straminicephala*, *Jurinea cataonica*)

A total of 15 *Klasea*, 1 *Serratula*, 1 *Jurinea* and 2 *Centaurea* taxa were used in the scoring analysis. The *Jurinea* and *Centaurea* taxa, which were used as the out-group, formed a cluster that was distinct from the *Klasea* and *Serratula* cluster in the constructed dendogram. Furthermore the *Klasea* and the *Serratula* taxa form clearly separate clusters among themselves (Figure 2).

The Klasea radiata subsp. radiata and Klasea radiata subsp. biebersteiniana taxa were observed to be very closely positioned in the dendogram. The *Klasea kotschyi*, *Klasea hakkiarica* and *Klasea haussknechtii* taxa, which have similar leaf characteristics, were also correlated in terms of their molecular data and were located in the same sub-cluster. *Klasea coriaceae* is a taxon, which spreads over distinct areas in the Eastern Anatolia and is distinguished owing to its distinctive height. The *Klasea oligocephala* and *Klasea kurdica* clustered together. The two taxa are very similar also in terms of morphological properties such as leaf, capitulum and pollen characteristics. *Klasea serratuloides* taxon has the largest capitulum among the genus. It has a similar profile to the *Klasea radiata* subspecies with respect to the leaf characteristics and the similarity of overspreading areas. *Klasea serratuloides* and *Klasea radiata* were also positioned close to one another on the dendogram.

*Klasea lasiocephala* is distinguished within the genus by its very short stems or the absent stems.. *Klasea lasiocephala* differs morphologically from other *Klasea* taxa in having absent or reduced stems and that it is also somewhat genetically distinct from other *Klasea* taxa, as the sole taxon in the cluster in which it is placed. The *Klasea erucifolia* and *Klasea grandifolia* taxa have similar leaf characteristics and were also located in the same sub-cluster owing to their molecular characteristics. *Klasea bornmuelleri* taxon does not have a morphologically close relative in the genus. Its position on the dendogram confirmed this classification. *Klasea cerinthifolia* is distinguished by its yellow flowers and semiamplexicaul leaf structure and was also molecularly identified to be distinct. All these findings were consistent with the morphological classifications made in the Flora of Turkey (Davis and Kupicha 1975; Dogan et al. 2012). Martins and Hellwig (2005) showed that *Klasea* and *Serratula* taxa to belong to separate clusters in a molecular study conducted using the ITS and ETS sequences. The same study reported shorter distances on the dendogram constructed based on molecular similarities for the taxa, which showed morphological similarities.

**Figure 3.**
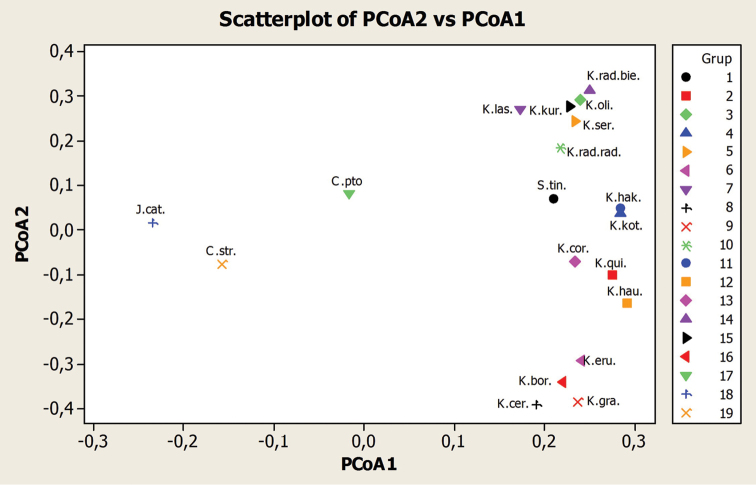
Principal co-ordinate analysis (PCA) of *Klasea*, *Serratula*, *Centaurea* and *Jurinea* species. **1** *Serratula tinctoria* **2** *Klasea quinquefolia* **3** *Klasea oligocephala* **4** *Klasea kotschyi* **5** *Klasea serratuloides* **6** *Klasea erucifolia* **7** *Klasea lasiocephala* **8** *Klasea cerinthifolia* **9** *Klasea grandifolia* **10** Klasea radiata subsp. radiata **11** *Klasea hakkiarica* **12** *Klasea haussknechtii* **13** *Klasea coriaceae* **14** Klasea radiata subsp. radiata **15** *Klasea kurdica* **16** *Klasea bornmuelleri* **17** *Centaurea ptosimopappoides* **18** *Centaurea straminicephala* **19** *Jurinea cataonica*

The inspection of the dendogram indicated that molecularly similar taxa were also morphologically similar. This separation was also shown in the PCA plot (Figure 3).

Our study has demonstrated that ISSR is a powerful tool in resolving the genetic relationships within problematic taxonomical entities. In conclusion, the morphologically close taxa were, in the molecular aspect, also located in the same clade. The genera used as out-groups (*Serratula*, *Jurinea*, and *Centaurea*) were clearly separate from the genus *Klasea*. According to our knowledge, this is the first report on the use of ISSR in *Klasea*.

## References

[B1] Ali MA Al-Hemaid FM Choudhary RK Lee J Kim SY Rub MA (2013) Status of *Reseda pentagyna* Abdallah & A.G. Miller (Resedaceae) inferred from combined nuclear ribosomal and chloroplast sequence data. Bangladesh Journal Plant Taxonomy 20(2): 233–238. doi: 10.3329/bjpt.v20i2.17397

[B2] APG (2003) An update of the Angiosperm Phylogeny Group classification for the orders and families of flowering plants: APG II. Botanical Journal of the Linnean Society 141: 399–436. doi: 10.1046/j.1095-8339.2003.t01-1-00158.x

[B3] Ash GJ Raman R Crump NS (2003) An investigation of genetic variation in *Carthamus lanatus* in New South Wales, Australia, using intersimple sequence repeats (ISSR) analysis. Weed Research 43: 208–213. doi: 10.1046/j.1365-3180.2003.00335.x

[B4] Barres L Sanmartin I Anderson CL Susanna A Buerki S Galbany-Casals M Vilatersana R (2013) Reconstructing the evolution and biogeographic history of tribe Cardueae (Compositae). American Journal of Botany 100(5): 867–882. doi: 10.3732/ajb.1200058 23624927 10.3732/ajb.1200058

[B5] Bremer K Backlund A Sennblad B Swenson U Andreasen K Hjertson M Lundberg J Backlund M Bremer B (2001) A phylogenetic analysis of 100+ genera and 50+ families of euasterids based on morphological and molecular data with notes on possible higher level morphological synapomorphies. Plant Systematics and Evolution 229: 137–169. doi: 10.1007/s006060170009

[B6] Davis PH Kupicha FK (1975) *Serratula* L. In: Davis PH (Ed.) Flora of Turkey and the East Aegean Islands, vol 5 Edinburgh Univ. Press, Edinburgh, 452–460.

[B7] Davis P Tan K Mill RR (Eds) (1988) Flora of Turkey and the East Aegean Islands, vol. 10 Edinburgh Univ. Press, Edinburgh.

[B8] Dogan B Duran A Hakki EE (2007) Phylogenetic analysis of *Jurinea* (Asteraceae) species from Turkey based on ISSR amplification. Annales Botanici Fennici 44: 353–358.

[B9] Dogan B Duran B Bagcı Y Dinc M Martin E Cetin Ö Oztürk M (2010) Phylogenetic relationships among the taxa of the genus *Johrenia* DC. (Apiaceae) from Turkey based on molecular method. Bangladesh Journal of Plant Taxonomy 17(2): 113–120. doi: 10.3329/bjpt.v17i2.6693

[B10] Dogan B Duran A Martin E Coşkun F (2012) Türkiye *Serratula* L. cinsinin revizyonu. Proje no: TÜBİTAK-TBAG-109T243 [In Turkish]

[B11] Fang DQ Roose ML Krueger RR Federici CT (1997) Fingerprinting of trifoliate orange germplasm accessions with isozymes, RFLPs, and inter-simple sequence repeat markers. Theoretical and Applied Genetics 95: 211–219. doi: 10.1007/s001220050550

[B12] Font M Garnatje T Garcia-Jacas N Susanna A (2002) Delineation and phylogeny of Centaurea sect. Acrocentron based on DNA sequences: a restoration of the genus *Crocodylium* and indirect evidence of introgression. Plant Systematics and Evolution 243: 15–26. doi: 10.1007/s00606-002-0203-3

[B13] Garcia-Jacas N Garnatje T Susanna A Vilatersana R (2002) Tribal and subtribal delimitation and phylogeny of the Cardueae (Asteraceae): A combined nuclear and chloroplast DNA analysis. Molecular Phylogenetics and Evolution 22(1): 51–64. doi: 10.1006/mpev.2001.1038 11796029 10.1006/mpev.2001.1038

[B14] Garcia-Jacas N Susanna A Garnatje T Vilatersana R (2001) Generic delimitation and phylogeny of the subtribe Centaureinae (Asteraceae): A combined nuclear and chloroplast DNA analysis. Annals of Botany 87: 503–515. doi: 10.1006/anbo.2000.1364

[B15] Garcia-Jacas N Susanna A Mozaffarian V Ilarslan R (2000) The natural delimitation of *Centaurea* (Asteraceae : Cardueae): ITS sequence analysis of the *Centaurea jacea* group. Plant Systematics and Evolution 223: 185–199. doi: 10.1007/BF00985278

[B16] Greuter W (2003) The Euro+Med treatment of Cardueae (Compositae) generic concepts and required new names. Willdenowia 33: 49–61. doi: 10.3372/wi.33.33104

[B17] Haffner E Hellwig FH (1999) Phylogeny of the Cardueae (Compositae) with emphasis on the subtribe Carduinae: an analysis based on ITS sequence data. Willdenowia 29: 27–39. doi: 10.3372/wi.29.2902

[B18] Hakki EE Savaskan C Akkaya MS (2001) Genotyping of Anatolian doubled-haploid durum lines with SSR markers. Euphytica 122: 257–262. doi: 10.1023/A:1012955516198

[B19] Hakki EE Akkaya MS (2000) Microsatellite isolation using amplified fragment length polymorphism markers: no cloning, no screening. Molecular Ecology 9: 2149–2154. doi: 10.1046/j.1365-294X.2000.11143.x 11123628

[B20] Joel D Portnoy V Tzuri G Greenberg R Katzir N (1998) Molecular markers of the identification of *Orobanche* species. Advance in Parasitic Plant Research Proceedings Sixth International Symposium on Parasitic Weeds, Cordoba, 152–160.

[B21] Jump AS Woodward FL Burke T (2003) *Cirsium* species show disparity in patterns of genetic variations at their range-edge, despite similar patterns of reproduction and isolation. New Phytologist 160: 359–370. doi: 10.1046/j.1469-8137.2003.00874.x 10.1046/j.1469-8137.2003.00874.x 33832174

[B22] Khalik KA El-Twab MA Gala R (2014) Genetic diversity and relationships among Egyptian *Galium* (Rubiaceae) and related species using ISSR and RAPD markers. Biologia 69(3): 300–310.

[B23] Laosatit K Tanta P Saensuk C Srinives P (2013) Development and characterization of EST-SSR markers from *Jatropha curcas* EST database and their transferability across jatropha-related species/genus. Biologia 68(1): 41–47. doi: 10.2478/s11756-012-0143-5

[B24] Martins L (2006) Systematics and Biogeography of *Klasea* (Asteraceae-Cardueae) and a synopsis of the genus. Botanical Journal of the Linnean Society 152: 435–465. doi: 10.1111/j.1095-8339.2006.00583.x

[B25] Martins L Hellwig FH (2005) Systematic position of the genere *Serratula* and *Klasea* within Centaureinae (Cardueae, Asteraceae) and new combinations in *Klasea*. Taxon 54: 632–638. doi: 10.2307/25065420

[B26] Mengitsu LW Messersmith CG (2002) Genetic diversity of kochia. Weed Science 50: 498–503. doi: 10.1614/0043-1745(2002)050[0498:GDOK]2.0.CO;2

[B27] Mohammadzadeh F Monirifar H Saba J Valizadeh M Haghighi AR Zanjani BM Barghi M Tarhriz V (2011) Genetic variation among Iranian Alfalfa (Medicago sativa L.) populations based on RAPD markers. Bangladesh Journal of Plant Taxonomy 18(2): 93–104. doi: 10.3329/bjpt.v18i2.9296

[B28] Nei M (1972) Genetic distance between populations. The American Naturalist 106: 283–292. doi: 10.1086/282771

[B29] Pharmawati M Yan G McFarlane IJ (2004) Application of RAPD and ISSR markers to analyse molecular relationships in *Grevillea* (Proteaceae). Australian Systematic Botany 17: 49–61. doi: 10.1071/SB03016

[B30] Prevost A Wilkinson MJ (1999) A new system of comparing PCR primers applied to ISSR fingerprinting of potato accessions. Theoretical and Applied Genetics 98: 107–112. doi: 10.1007/s001220051046

[B31] Rohlf FJ (1992) NTSYS-pc: Numerical taxonomy and multivariate analysis system, version 2.0. State Univ. of New York, Stony Brook, NY.

[B32] Soranzo N Provan J Powel W (1999) An example of microsatellite length variation in the mitochondrial genome of conifers. Genome 42: 158–161. doi: 10.1139/g98-111 10208008

[B33] Susanna A Garcia-Jacas N Hidalgo O Vilatersana R Garnatje T (2006) The Cardueae (Compositae) Revisited: Insights from ITS, Trnl-Trnf, and Matk Nuclear and Chloroplast DNA Analysis. Annals of the Missouri Botanical Garden 93: 150–171. doi: 10.3417/0026-6493(2006)93[150:TCCRIF]2.0.CO;2

[B34] Vosman B Arens P (1997) Molecular characterization of GATA/GACA microsatellite repeat in tomato. Genome 40: 25–33. doi: 10.1139/g97-004 9061911 10.1139/g97-004

[B35] Williams JGK Kubelik AR Livak KJ (1990) DNA polymorphism amplified by arbitrary primers are useful as genetic markers. Nucleic Acids Research 18: 6531–6535. doi: 10.1093/nar/18.22.6531 1979162 10.1093/nar/18.22.6531 PMC332606

[B36] Wolfe AD Qiu-Yun X Kepkart SR (1998) Assessing hybridization in natural populations of *Penstemon* (Scrophulariaceae) using hypervariable intersimple sequence repeat (ISSR) bands. Molecular Ecology 7: 1107–1125. doi: 10.1046/j.1365-294x.1998.00425.x 9734070 10.1046/j.1365-294x.1998.00425.x

[B37] Yang W De Oliveria AC Godwin I Schertz K Bennetzen JL (1996) Comparison of DNA marker technologies in characterizing plant genome diversity: variability of Chinese sorghums. Crop Science 36: 1669–1676. doi: 10.2135/cropsci1996.0011183X003600060042x

[B38] Zietkiewicz E Rafalski A Labuda D (1994) Genome fingerprinting by simple sequence repeat (SSR)-anchored polymerase chain reaction amplification. Genomics 20: 176–183. doi: 10.1006/geno.1994.1151 8020964 10.1006/geno.1994.1151

